# Interactions between *FLORAL ORGAN NUMBER4* and floral homeotic genes in regulating rice flower development

**DOI:** 10.1093/jxb/erw459

**Published:** 2017-01-20

**Authors:** Wei Xu, Juhong Tao, Mingjiao Chen, Ludovico Dreni, Zhijing Luo, Yun Hu, Wanqi Liang, Dabing Zhang

**Affiliations:** 1 Joint International Research Laboratory of Metabolic & Developmental Sciences, State Key Laboratory of Hybrid Rice, Shanghai Jiao Tong University–University of Adelaide Joint Centre for Agriculture and Health, School of Life Sciences and Biotechnology, Shanghai Jiao Tong University, Shanghai, China; 2 Instituto de Biología Molecular y Celular de Plantas, Consejo Superior de Investigaciones Científicas-Universidad Politécnica de Valencia, Valencia, Spain; 3 School of Agriculture, Food and Wine, University of Adelaide, Waite Campus, Urrbrae, SA, Australia

**Keywords:** Floral homeotic genes, floral meristem, flower development, *FON4*, genetic interaction, rice

## Abstract

The floral meristem (FM) is self-maintaining at the early stages of flower development, but it is terminated when a fixed number of floral organs are produced. The *FLORAL ORGAN NUMBER4* (*FON4*; also known as *FON2*) gene, an ortholog of Arabidopsis *CLAVATA3* (*CLV3*), is required for regulating FM size and determinacy in rice. However, its interactions with floral homeotic genes remain unknown. Here, we report the genetic interactions between *FON4* and floral homeotic genes *OsMADS15* (an A-class gene), *OsMADS16* (also called *SUPERWOMAN1*, *SPW1*, a B-class gene), *OsMADS3* and *OsMADS58* (C-class genes), *OsMADS13* (a D-class gene), and *OsMADS1* (an E-class gene) during flower development. We observed an additive phenotype in the *fon4* double mutant with the *OsMADS15* mutant allele *dep* (*degenerative palea*). The effect on the organ number of whorl 2 was enhanced in *fon4 spw1*. Double mutant combinations of *fon4* with *osmads3*, *osmads58*, *osmads13*, and *osmads1* displayed enhanced defects in FM determinacy and identity, respectively, indicating that *FON4* and these genes synergistically control FM activity. In addition, the expression patterns of all the genes besides *OsMADS13* had no obvious change in the *fon4* mutant. This work reveals how the meristem maintenance gene *FON4* genetically interacts with C, D, and E floral homeotic genes in specifying FM activity in monocot rice.

## Teaser

Rice flower development determines grain yield. Here we reveal the genetic interactions between the rice meristem maintenance gene *FON4* and six floral homeotic genes in flower development.

## Introduction

Plants possess the ability to produce organs throughout their life due to the continuous activity of meristems. Maintenance of meristem activity is dependent on the balance between differentiation and self-renewal of stem cells located in the central zone (Steeves and Sussex, 1989). In the eudicot *Arabidopsis thaliana*, the feedback loop consisting of the homeodomain transcription factor WUSCHEL (WUS) and the CLAVATA (CLV) ligand–receptor plays a prominent role in the stem cell maintenance of the shoot apical meristem (SAM) and floral meristem (FM) (Fletcher *et al.*, 1999; Brand *et al.*, 2000; Schoof *et al.*, 2000; Carles and Fletcher, 2003; Aichinger *et al.*, 2012; Perales and Reddy, 2012). *WUS* is expressed in the organizing center (OC) of meristems and migrates into overlying cells of the central zone (CZ), where it specifies stem cell fate and activates a small secreted peptide CLV3 (Mayer *et al.*, 1998; Schoof *et al.*, 2000; Yadav *et al.*, 2011; Daum *et al.*, 2014). CLV3 activates receptor kinase signaling and, in turn, restricts WUS activity (Fletcher *et al.*, 1999; Brand *et al.*, 2000; Schoof *et al.*, 2000). To date, there are at least four receptors known to be required for perceiving CLV3 peptide, including the leucine-rich repeat (LRR) receptor-like kinase CLV1 (Clark *et al.*, 1997; Ogawa *et al.*, 2008), the LRR receptor-like protein CLV2 (Jeong *et al.*, 1999), the pseudokinase CORYNE (CRN)/SUPPRESSOR OF LLP1 2(SOL2) (Müller *et al.*, 2008; Miwa *et al.*, 2008; Bleckmann *et al.*, 2010; Nimchuk *et al.*, 2011), and RECEPTOR-LIKE PROTEIN KINASE 2 (RPK2)/TOADSTOOL 2 (TOAD2) (Kinoshita *et al.*, 2010). Through the feedback regulatory loop between *WUS* and *CLV*, the stem cell population within meristems is maintained at a relatively constant number.

Unlike the indeterminate SAM, the FM ceases to have stem cell activity after the formation of a certain number of floral organs (Steeves and Sussex, 1989), whose identities have been proposed to be specified by A, B, C, D, and E floral homeotic genes. This is described by the ‘ABCDE’ model which is based on the research in model eudicot plants (Coen and Meyerowitz, 1991; Angenent *et al.*, 1995; Colombo *et al.*, 1995; Pelaz *et al.*, 2000; Theißen, 2001; Theissen and Saedler, 2001; Ditta *et al.*, 2004; Krizek and Fletcher, 2005). In *A. thaliana*, the ABCDE model includes floral organ identity genes which are also responsible for the specification or termination of the FM, such as the A-class genes *APETALA1* (*AP1*) and *APETALA2* (*AP2*), the C-class gene *AGAMOUS* (*AG*), and the four E-class genes *SEPALLATA1* (*SEP1*), *SEP2*, *SEP3*, and *SEP4* (Irish and Sussex, 1990; Yanofsky *et al.*, 1990; Mandel *et al.*, 1992; Jofuku *et al.*, 1994;  Pelaz *et al.*, 2000; Lenhard *et al.*, 2001; Lohmann *et al.*, 2001; Ditta *et al.*, 2004). Among them, the C-class gene *AG* plays a major role in the termination of the FM, beside its function in specifying stamen and carpel identities (Yanofsky *et al.*, 1990; Lenhard *et al.*, 2001; Lohmann *et al.*, 2001). Furthermore, at the early floral developmental stage, *WUS*, together with the meristem identity gene *LEAFY* (*LFY*), activates *AG* expression in the inner two whorls (Lenhard *et al.*, 2001; Lohmann *et al.*, 2001). At the later stage, *AG* represses *WUS* activity through two independent mechanisms: the activation of *KNUCKLES* (*KNU*), a *WUS* repressor, and via the recruitment of Polycomb Group (PcG) proteins to *WUS* (Ming and Ma, 2009; Sun *et al.*, 2009, 2014; Liu *et al.*, 2011; Zhang, 2014). Although both *AG* and the *CLV* pathways negatively regulate *WUS* expression, *CLV* genes and *AG* appear to repress *WUS* independently, as the phenotype of *clv1 ag* double mutants was substantially additive and the *WUS* expression domain was larger in *clv1 ag* double mutants than in *ag* single mutants (Clark *et al.*, 1993; Lohmann *et al.*, 2001).

In grasses, the basic structural unit of the inflorescence is called the spikelet, and each consists of glumes and one or several florets (Zhang *et al.*, 2013; Zhang and Yuan, 2014; Yuan and Zhang, 2015). Normally, each floret contains two bract-like organs (lemma and palea), lodicules, stamens, and a pistil (Kellogg, 2001; Rudall and Bateman, 2004; Yuan *et al.*, 2009). The lodicules are considered to be the counterparts to the eudicot petals, whereas the origin of the palea and the lemma still remains controversial. Inflorescence and flower development in the grass species are markedly distinct from those in eudicots. Nevertheless, genetic studies on two model plants, maize (*Zea mays* L.) and rice (*Oryza sativa* L.), have demonstrated that the *CLV* signaling pathway of meristem maintenance and the ABCDE model of floral organ specification are partially conserved between grasses and eudicots (Ferrario *et al.*, 2004; Bommert *et al.*, 2005*b*; Thompson and Hake, 2009; Ciaffi *et al.*, 2011; Pautler *et al.*, 2013; Tanaka *et al.*, 2013; Zhang *et al.*, 2013; Zhang and Yuan, 2014; Wang *et al*., 2015; Dreni and Zhang, 2016).

In maize, mutations in *THICK TASSEL DWARF1* (*TD1*) and *FASCIATED EAR2* (*FEA2*) genes, which encode orthologs of *CLV1* and *CLV2*, respectively, affect the size of the inflorescence meristem and FM (Taguchi-Shiobara *et al.*, 2001; Bommert *et al.*, 2005*a*). In addition, *TD1* and *FEA2* function in different pathway since the *td1 fea2* double mutant shows an additive or synergistic phenotype (Bommert *et al.*, 2005*a*). This may imply a different mechanism in maize, because *CLV1* and *CLV2* were once thought to act in a common pathway in Arabidopsis (Kayes and Clark, 1998). However, subsequent evidence has revealed that CLV2 functions separately from CLV1 by forming heteromers with CRN/SOL2 (Müller *et al.*, 2008; Miwa *et al.*, 2008; Bleckmann *et al.*, 2010; Nimchuk *et al.*, 2011). Apart from these two *CLV*-like genes, maize *COMPACT PLANT2* (*CT2*) encoding the α-subunit (Gα) of a heterotrimeric GTP-binding protein, was also shown to be directly involved in the *CLV* pathway. Biochemical and genetic analyses indicate that CT2/Gα has an interaction with FEA2 in controlling meristem development (Bommert *et al.*, 2013).

In rice, *FLORAL ORGAN NUMBER1* (*FON1*) and *FON4* (also known as *FON2*) are closely related to *CLV1* and *CLV3*, respectively. Both *fon1* and *fon4* mutants have enlarged FMs, and an increased floral organ number, especially stamens and pistils (Suzaki *et al.*, 2004，^2006^; Chu *et al.*, 2006). Moreover, it has been shown that *FON4* and *FON1* function in a common pathway in specifying FM maintenance, mimicking that of *CLV3* and *CLV1* (Chu *et al.*, 2006; Suzaki *et al.*, 2006; Chu and Zhang, 2007).

Taken together, these studies suggest that the *CLV* pathway is conserved in the regulation of meristem size between eudicot and grass species. However, there are some differences between *CLV* genes and corresponding grass orthologs in expression patterns and mutant phenotypes. *CLV1* expression is mainly detected in the L3 layers of meristems (Clark *et al.*, 1997; Fletcher *et al.*, 1999), whereas *TD1* and *FON1* are uniformly expressed throughout the meristems, as well as ﬂoral organ primordia (Suzaki *et al.*, 2004; Bommert *et al.*, 2005*a*). Unlike the *clv1* mutant, which displays enlargement of the inflorescence meristem and FM, the rice *fon1* mutant has no evident defect in inflorescence meristem size, although it produces an enlarged FM.

In grass species, *AP1*-like genes are required for the phase transition from vegetative to reproductive growth, such as *VRN1* of *Triticum monococcum* or *WAP1* of *T. aestivum* (Murai *et al.*, 2003; Yan *et al.*, 2003), which differs from Arabidopsis *AP1* with a role in establishing FM identity and specifying the identities of the outer two whorls, the sepal and petal (Mandel *et al.*, 1992). The rice genome has four *AP1*-like MADS-box genes, *OsMADS14* (*RAP1B*), *OsMADS15* (*RAP1A*), *OsMADS18*, and *OsMADS20* (Litt and Irish, 2003; Kater *et al.*, 2006). On the basis of their expression pattern and phenotypic analyses on available mutants or transgenic plants, it was proposed that rice *AP1*-like genes have a function in FM identity specification (Jeon *et al.*, 2000*b*; Kyozuka *et al.*, 2000; Masiero *et al.*, 2002; Pelucchi *et al.*, 2002; Lee *et al.*, 2003，^2004^; Fornara *et al.*, 2004; Komiya *et al.*, 2008; Wang *et al.*, 2010; Taoka *et al.*, 2011; Lu *et al.*, 2012). In contrast, B-class MADS-box genes have conserved functions in both eudicots and grasses. Rice *OsMADS16* (also called *SUPERWOMAN1*, *SPW1*) and maize *Silky1*, two orthologs of the Arabidopsis B-function gene *APETALA3* (*AP3*), are both essential for determining lodicule and stamen identity (Ambrose *et al.*, 2000; Nagasawa *et al.*, 2003; Whipple *et al.*, 2007). Genetic analyses also reveal that *OsMADS16* and *Silky1* are involved in the control of the FM determinacy together with C-class genes (Ambrose *et al.*, 2000; Yun *et al.*, 2013). A recent work suggested that, in maize, the *PI/GLO*-like B-class genes may also have a similar function (Bartlett *et al.*, 2015). C-class genes have been partially subfunctionalized by means of gene duplication during grass evolution (Kramer *et al.*, 2004; Zahn *et al.*, 2006; Dreni *et al.*, 2013; Dreni and Kater, 2014). In rice, two C-class genes, *OsMADS3* and *OsMADS58*, redundantly regulate the identity of reproductive organs and FM determinacy (Yamaguchi *et al.*, 2006; Dreni *et al.*, 2011; Hu *et al.*, 2011). Likewise, maize has three *AG* orthologs: *ZAG1* (*ZEA AGAMOUS1*), *ZMM2* (*ZEA MAYS MADS2*), and *ZMM23* (Schmidt *et al.*, 1993; Mena *et al.*, 1996; Münster *et al.*, 2002). In the *zag1* mutant, FM partially lost determinacy, but the identity of reproductive organs was almost normal, suggesting that other class C genes may be required for stamen and carpel specification, such as *ZMM2* and *ZMM23*, whose functions remain unknown to date (Schmidt *et al.*, 1993; Mena *et al.*, 1996; Münster *et al.*, 2002). In addition, rice *OsMADS13*, one ortholog of Arabidopsis *SEEDSTICK* (*STK*) and petunia *FLORAL BINDING PROTEIN7* (*FBP7*) and *FBP11* D-class genes, controls ovule identity and FM determinacy (Angenent *et al.*, 1995; Colombo *et al.*, 1995; Pinyopich *et al.*, 2003; Dreni *et al.*, 2007, 2011; Li *et al.*, 2011*b*). However, the Arabidopsis *stk* single mutant does not display the conversion of ovule identity because of functional redundancy with *AG*, *SHATTERPROOF1* (*SHP1*), and *SHP2* (Favaro *et al.*, 2003; Pinyopich *et al.*, 2003). A number of *SEP* subfamily (E-class) genes with diverse function have been identified from grasses. There are five members [*OsMADS1/LEAFY HULL STERILE1* (*LHS1*)/*NAKED SEED RICE* (*NSR*), *OsMADS5*, *OsMADS7*, *OsMADS8*, and *OsMADS34*] in rice and eight in maize (Malcomber and Kellogg, 2005; Zahn *et al.*, 2005). Rice *OsMADS1* was shown to determine lemma and palea identity and to promote FM specification by co-ordinating transcriptional control and hormone signaling pathways (Jeon *et al.*, 2000*a*; Prasad *et al.*, 2001, 2005; Agrawal *et al.*, 2005; Chen *et al.*, 2006; Gao *et al.*, 2010; Li *et al.*, 2010; Khanday *et al.*, 2013; Hu *et al.*, 2015).

To reveal whether *FON4* interacts with floral homeotic genes in specifying rice flower development, we constructed and analyzed the double mutants of *FON4* with *OsMADS15*, *OsMADS16*, *OsMADS3*, *OsMADS58*, *OsMADS13*, and *OsMADS1*, respectively. Therefore, we concluded that *FON4* and C, D, and E floral homeotic genes play a synergistic role in specifying FM activity and flower development. This work provides insight into the mechanism controlling FM activity in rice.

## Materials and methods

### Plant materials

In this study, we used the rice (*Oryza sativa*) mutants *fon4-2*, *fon4-1*, *dep* (*degenerative palea*), *spw1-1*, *osmads3-4*, *osmads58*, *osmads13-3*, and *osmads1-z*. The *fon4-2*, *fon4-1*, *osmads3-4*, *osmads58*, *osmads13-3*, and *osmads1-z* mutants were previously reported (Chu *et al.*, 2006; Gao *et al.*, 2010; Dreni *et al.*, 2011; Hu *et al.*, 2011; Li *et al.*, 2011*b*). *dep* was kindly provided by Professor Zhukuan Cheng (Chinese Academy of Sciences), and *spw1-1* was provided by Professor Hajime Sakai and Professor Yasuo Nagato (University of Tokyo). Double mutants were isolated by genotyping and phenotype observation. Primers for genotyping are listed in Supplementary Table S1 at *JXB* online. All the mutants and wild-type rice (9522 cultivar) were grown in the paddy field or greenhouse of Shanghai Jiao Tong University, China.

### Histological analysis and microscopy observation

Fresh spikelets were photographed with a Leica S8 APO stereo microscope. For histological analysis, samples were prepared and observed following the method reported by Hu *et al.* (2015). Scanning electron microscopy (SEM) observations were performed as described previously (Li *et al.*, 2006). Images were processed through Adobe Photoshop CS6 software.

### *In situ* hybridization

The inflorescences of wild-type rice plants were fixed overnight at 4 °C in FAA (5% acetic acid, 50% ethanol, and 3.7% formaldehyde in water), dehydrated in an ethanol series, and embedded in Paraplast Plus (Sigma). The hybridization signals were detected according to the previous description (Chu *et al.*, 2006). The probes for *OsMADS15*, *OsMADS16, OsMADS3, OsMADS58, OsMADS13, OsMADS1*, and *OSH1* were prepared as previously reported (Li *et al.*, 2010, 2011*a*, *b*).

### qRT–PCR

Total RNAs were extracted with TRIZOL reagent (Sigma-Aldrich), and ~1 μg of RNA was reverse transcribed using the PrimeScript RT reagent kit with genomic DNA eraser (DRR047A; Takara). The 10-fold diluted cDNA samples were used as templates for the quantitative reverse transcription–PCR (qRT–PCR) experiment. The qRT–PCRs were performed on a Bio-Rad CFX96 Real-Time System using the iQ SYBR Green Supermix (Bio-Rad). The amplifying program was as follows: 95 °C for 30 s, 40 cycles of 95 °C for 5 s and 60 °C for 30 s each. Three biological replicates were conducted with three technical replicates each, and the relative expression levels of the genes were quantified using a relative quantitation method (Δ cycle threshold). Data were normalized by the reference gene *ACTIN* (LOC_Os03g50885.1). Primers for qRT–PCR analyses are listed in Supplementary Table S1.

## Results

### *FON4* and *OsMADS15* function in different pathways

**Fig. 1.**
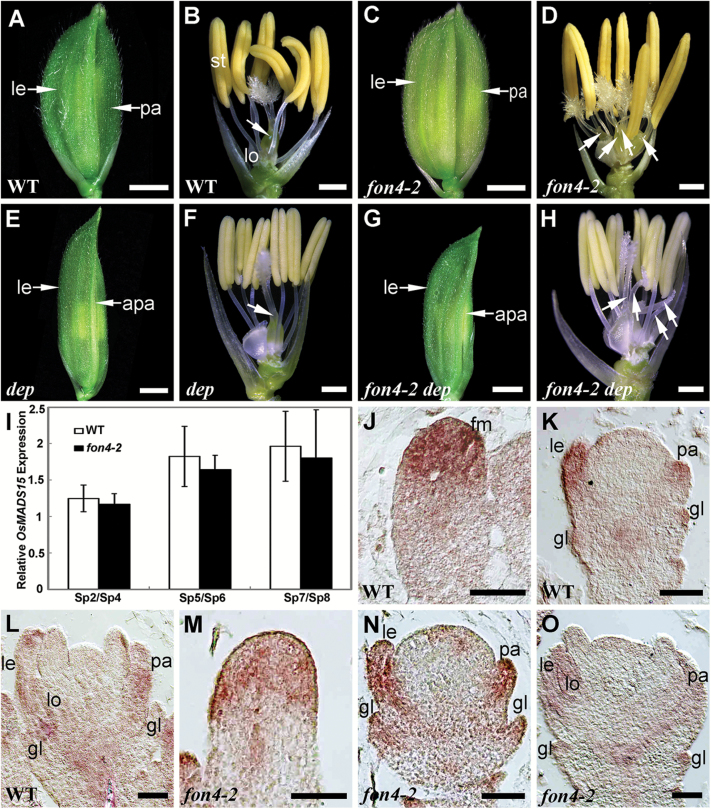
Phenotype of *fon4-2 dep*. (A, B) Wild-type (WT) flower with one lemma, one palea, two lodicules, six stamens, and one pistil. (C, D) *fon4-2* flower with one lemma, one palea, two lodicules, seven stamens, and four pistils. (E, F) *dep* flower with one lemma, one abnormal palea, two lodicules, six stamens, and one pistil. (G, H) *fon4-2 dep* flower with one lemma, one abnormal palea, two lodicules, six stamens, and four pistils. The lemma and palea were removed in (B, D, F, H), and arrows indicate pistils. (I) qRT–PCR analysis of *OsMADS15* expression in wild-type and *fon4-2* flowers at stage Sp2/Sp4, Sp5/Sp6, and Sp7/Sp8. (J–O) *OsMADS15* expression pattern in wild-type (J–L) and *fon4-2* (M–O) flowers at different stages of flower development. apa, abnormal palea; fm, floral meristem; gl, glume; le, lemma; lo, lodicule; pa, palea; st, stamen. Scale bars=2 mm (A, C, E, G), 1 mm (B, D, F, H), and 50 μm (J–O).

As a result of FM determinacy, a wild-type rice floret consists of a fixed number of floral organs, including two leaf-like organs, the lemma and palea, two lodicules, six stamens, and one pistil from the outer to inner whorls (Fig. 1A, B; Table 1) (Yuan *et al.*, 2009; Zhang *et al.*, 2013). Mutations in *FON4* cause an increase in the number of floral organs, especially stamens and pistils (Chu *et al.*, 2006; Suzaki *et al.*, 2006). The *fon4-2* allele, which contains a G-to-A base alteration at the 3' end of the first intron of *FON4*, generated 6–8 stamens and 2–4 pistils, whereas the number of paleas, lemmas, and lodicules showed almost no change (Fig. 1C, D; Table 1) (Chu *et al.*, 2006)*. OsMADS15*, an ortholog of Arabidopsis *AP1*, is required for flowering time regulation and palea identity specification during rice flower development. The *OsMADS15* mutant, *dep*, displays shrunken paleas, and weak defects in lemmas and glumes (Fig. 1E, F; Table 1) (Wang *et al.*, 2010). To examine whether *FON4* has a genetic interaction with *OsMADS15*, a *fon4-2 dep* double mutant was created by genetic crosses. The *fon4-2 dep* double mutant exhibited additive phenotypes of the two single mutants, as both the abnormal paleas and the increased number of floral organs were observed (Fig. 1G, H; Table 1). Furthermore, we compared the expression pattern of *OsMADS15* in the wild type and *fon4-2* during flower development. The qRT–PCR result showed that *OsMADS15* expression was not obviously changed in the *fon4-2* mutant from stage Sp2 to stage Sp8 (Fig. 1I) (the stages defined by Ikeda *et al.*, 2004). Consistently, the expression pattern of *OsMADS15* in *fon4-2* was similar to that in the wild type via *in situ* hybridization analysis. In the flowers of both the wild type and *fon4-2*, *OsMADS15* was initially expressed in the apical region of the FM at the early stage of flower development (Fig. 1J, M). When the lemma and palea primordia were formed, *OsMADS15* expression was detected in the glumes, paleas, and lemmas (Fig. 1K, N). After the stamen primordia initiated, *OsMADS15* was expressed in glumes, lemmas, paleas, and lodicules (Fig. 1L, O; Supplementary Fig. S1) (Kyozuka *et al.*, 2000). Thus, we proposed that *FON4* and *OsMADS15* function in parallel pathways in specifying flower development.

**Table 1.** The number of floral organs in the wild type and mutants

Genotype	No. of spikelets examined	Lemma/ palea	Glume- like organs	Normal lodicules	Normal stamens	Lodicule- like organs	Fused gynoecia	Stigmas
Wild type	20	2	0	2	6	0	1	2
*fon4-2*	103	2	0	2.16 ± 0.39	6.46 ± 0.75	0	2.96 ± 1.03	7.04 ± 2.25
*dep*	20	2	0	2	6	0	1	2
*fon4-2 dep*	97	2	0	2.32 ± 0.59	6.10 ± 0.55	0	2.44 ± 0.82	5.41 ± 1.70
*spw1-1*	56	2	2.21 ± 0.46	0	0	0	7.71 ± 1.11	16.52 ± 2.17
*fon4-2 spw1-1*	57	2	3.49 ± 0.91	0	0	0	7.77 ± 1.52	20.40 ± 3.88
*osmads3-4*	100	2	0	2.30 ± 0.63	4.94 ± 1.39	1.30 ± 1.57	1.06 ± 0.33	2.13 ± 0.77
*fon4-2 osmads3-4*	73	2	0	3.66 ± 1.52	0.12 ± 0.44	6.15 ± 0.83	7.89 ± 2.11	18.73 ± 5.17
*fon4-1*	100	2.18 ± 0.38	0	2.51 ± 0.62	6.44 ± 0.59	0	2.86 ± 0.78	6.08 ± 1.94
*osmads58*	109	2	0	2	6	0	1	2.25 ± 0.45
*fon4-1 osmads58*	102	2.16 ± 0.37	0	2.52 ± 0.66	6.48 ± 1.14	0	4.53 ± 1.19	8.11 ± 1.97
*osmads13-3*	150	2	0	2	6	0	1	2.19 ± 0.42
*fon4-1 osmads13-3*	100	2.09 ± 0.29	0	2.30 ± 0.55	6.35 ± 0.67	0	7.98 ± 1.97	19.84 ± 4.93

The average number is shown as the mean ±SD.

### *FON4* acts in parallel with *OsMADS16* in specifying flower development

**Fig. 2.**
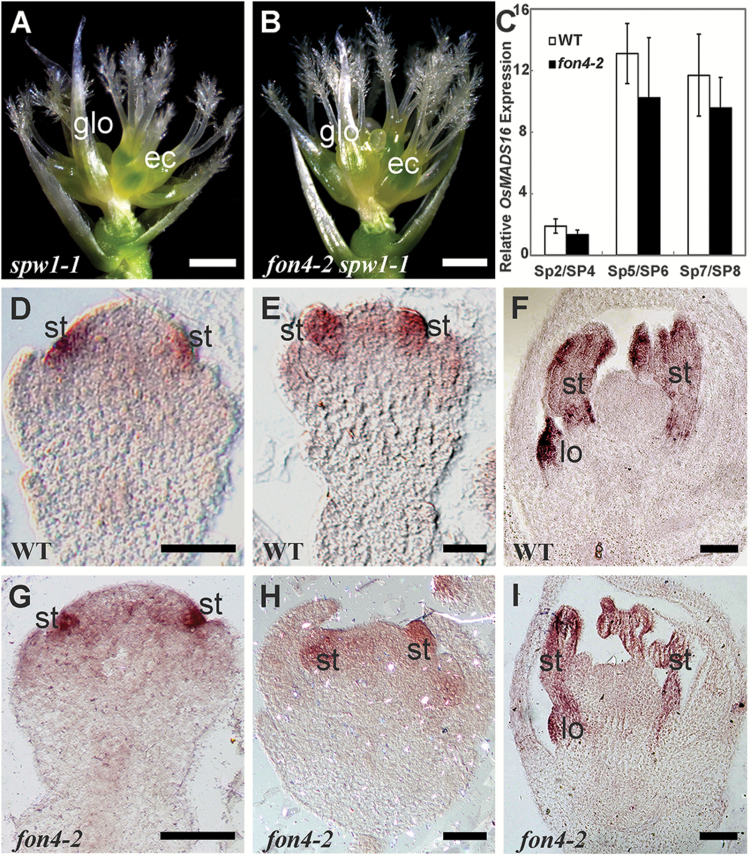
Phenotype of *fon4-2 spw1-1*. (A) *spw1-1* flower with glume-like organs and ectopic carpels. (B) *fon4-2 spw1-1* flower. The palea and lemma were removed in (A) and (B). (C) qRT–PCR analysis of *OsMADS16* expression in wild-type and *fon4-2* flowers. (D–I) *OsMADS16* expression pattern in wild-type (D–F) and *fon4-2* (G–I) flowers. ec, ectopic carpel; glo, glume-like organ; lo, lodicule; st, stamen. Scale bars=1 mm (A, B) and 50 μm (D–I).

The rice B-class gene *OsMADS16* was shown to specify the identity of lodicules and stamens (Nagasawa *et al.*, 2003; Li *et al.*, 2011*a*; Yun *et al.*, 2013). *spw1-1* flowers display the conversion of lodicules and stamens into glume-like organs and carpel-like organs, respectively (Fig. 2A; Table 1) (Li *et al.*, 2011*a*; Yun *et al.*, 2013). Our recent discoveries revealed that *OsMADS16* is also involved in the control of the FM determinacy together with C-class genes *OsMADS3* and *OsMADS58* or the E-class gene *OsMADS6* (Li *et al.*, 2011*a*; Yun *et al.*, 2013). We observed that the number of glume-like organs in whorl 2 of the *fon4-2 spw1-1* double mutant showed a distinct increase compared with *spw1-1* (Fig. 2B; Table 1). Since the *fon4-2* mutant only exhibited a slight change in the number of lodicules, there may be a synergistic interaction between *FON4* and *OsMADS16* in whorl 2. On the other hand, the number of fused gynoecia and stigmas in the inner whorls had no apparent change in *fon4-2 spw1-1* compared with *spw1-1*, suggesting that *FON4* may have a less obvious interaction with *OsMADS16* in specifying the inner flower organ development. To test whether *OsMADS16* and *FON4* have a transcriptional regulatory relationship, we performed qRT–PCR and *in situ* hybridization (Fig. 2C–I). In the wild-type flower, *OsMADS16* expression was first detected in the incipient lodicule and stamen primordia, and then in the developing lodicule and stamen primordia (Fig. 2D–F; Supplementary Fig. S1) (Nagasawa *et al.*, 2003; Yun *et al.*, 2013). Analogously, we observed that *OsMADS16* expression in *fon4-2* was restricted to the two floral organs (Fig. 2G–I). Collectively, we concluded that *FON4* and *OsMADS16* synergistically regulate the organ number of the whorl 2, and they might function independently during flower development.

### Interactions of *FON4* with *OsMADS3* and *OsMADS58*

**Fig. 3.**
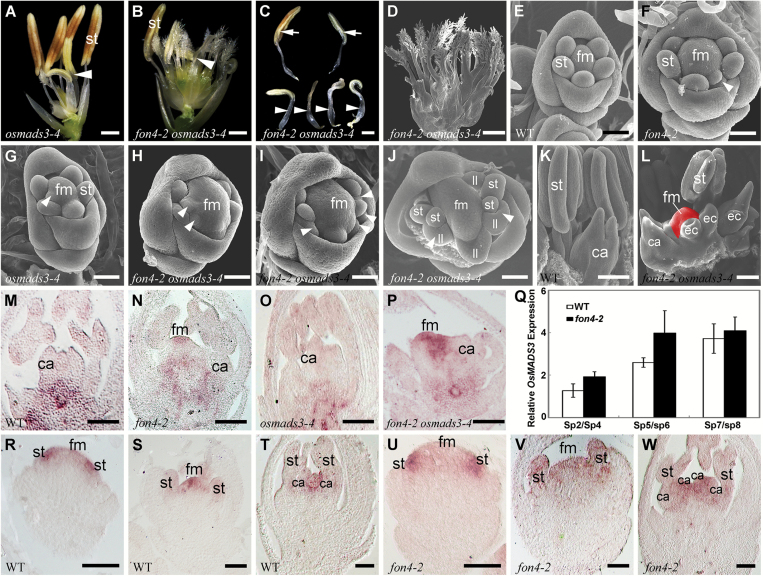
Phenotype of *fon4-2 osmads3-4*. (A) *osmads3-4* flower. (B) *fon4-2 osmads3-4* flower. The lemma and palea were removed in (A) and (B), and arrowheads indicate lodicule-like organs. (C) A close-up of normal stamens (arrows) and lodicule-like organs (arrowheads) in (B). (D) SEM observation of numerous carpels in *fon4-2 osmads3-4*. (E–J) SEM images of wild-type (E), *fon4-2* (F), *osmads3-4* (G), and *fon4-2 osmads3-4* (H–J) flowers at the stage when stamen primordia are formed in whorl 3. Arrowheads in (F–J) indicate organ primordia developed from the additional whorl. A partial palea was removed in (J). (K) SEM image of a wild-type flower at the stage when the pistil with two stigmas is produced. (L) SEM image of a *fon4-2 osmads3-4* flower at the stage that corresponds to that of (K). The floral meristem (red region) remains in *fon4-2 osmads3-4* even after several carpels have been formed. (M–P) *OSH1* expression in wild-type (M), *fon4-2* (N), *osmads3-4* (O), and *fon4-2 osmads3-4* (P) flowers after the formation of carpels. *OSH1* expression completely disappears when the carpels are formed in the wild-type and *osmads3-4* flowers, whereas it continues to be expressed in the FM around the carpel primordia in *fon4-2* and *fon4-2 osmads3-4* flowers. Moreover, the *OSH1* expression region is larger in *fon4-2 osmads3-4* than in the *fon4-2* single mutant. (Q) qRT–PCR analysis of *OsMADS3* expression in wild-type and *fon4-2* flowers. (R–W) *OsMADS3* expression pattern in wild-type (R–T) and *fon4-2* (U–W) flowers. ca, carpel; ec, ectopic carpel; fm, floral meristem; ll, lodicule-like organ; st, stamen. Scale bars=1 mm (A–C), 500 μm (D), 50 μm (E–J, R–W), and 100 μm (K–P).

The rice genome contains two duplicated C-class genes, *OsMADS3* and *OsMADS58*, which redundantly regulate reproductive organ identity and FM determinacy (Yamaguchi *et al.*, 2006; Dreni *et al.*, 2011). In the *osmads3-4* mutant, an intermediate allele of *OsMADS3*, the stamens in whorl 3 were partially converted into lodicule-like organs, whereas the carpel in whorl 4 developed almost normally (Fig. 3A; Table 1) (Hu *et al.*, 2011). *fon4-2 osmads3-4* showed additive effects on the number of floral organs in whorl 3, but the number of lodicule-like organs that transformed from stamens was significantly increased in the double mutant compared with that of the *osmads3-4* mutant (Fig. 3B, C; Table 1), suggesting that *fon4-2* enhances the stamen identity defect seen in *osmads3-4.* Moreover, the *fon4-2 osmads3-4* double mutant displayed dramatically enhanced defects of FM determinacy, where more carpel-like structures were produced than either of the single mutants (Fig. 3D; Table 1).

Furthermore, our SEM analysis revealed that at the stage when the stamen primordia were formed in whorl 3 of the wild-type flower (Fig. 3E), *fon4-2* showed an increased size of the FM, and extra stamens in the same whorl or in an additional whorl compared with the wild type (Fig. 3F), and *osmads3-4* generated extra organ primordia with an irregular shape inside whorl 3, reflecting a homeotic transformation of stamens (Fig. 3G). *fon4-2 osmads3-4* exhibited a more severe homeotic conversion of stamens, indicated by fewer normal stamen primordia in whorl 3 and more abnormal primordia detected in an additional whorl compared with the *osmads3-4* single mutant. The double mutant also had a dramatically enlarged FM compared with that of either single mutant (Fig. 3H–J; Supplementary Table S2). After the formation of carpel primordium in whorl 4, the FM activity was terminated in the wild type (Fig. 3K), but the FM of *fon4-2 osmads3-4* appeared to be still active, even after a number of carpels had formed (Fig. 3L). Furthermore, *in situ* analysis showed that *OSH1*, a molecular marker of undifferentiated cells in rice meristems (Sato *et al.*, 1996), continued to be expressed in the central region after several ectopic carpels formed in *fon4-2 osmads3-4* flowers, whereas *OSH1* expression disappeared after the formation of carpels in the wild type (Fig. 3M, P). In addition, the region of *OSH1* expression was larger in the double mutant than in either of the single mutants (Fig. 3N–P). These results suggest that *FON4* and *OsMADS3* synergistically regulate reproductive organ identity and FM determinacy, and loss of function of both genes causes an enlarged FM. To dissect further the relationship between *FON4* and *OsMADS3*, we performed qRT–PCR assays and observed that there was no obvious expression change of *OsMADS3* in *fon4-2* from stage Sp2 to stage Sp8 (Fig. 3Q). Consistently, *in situ* hybridization analysis showed that *OsMADS3* expression in *fon4-2* mirrored that in the wild type (Fig. 3R–W; Supplementary Fig. S1) (Dreni *et al.*, 2011).

**Fig. 4.**
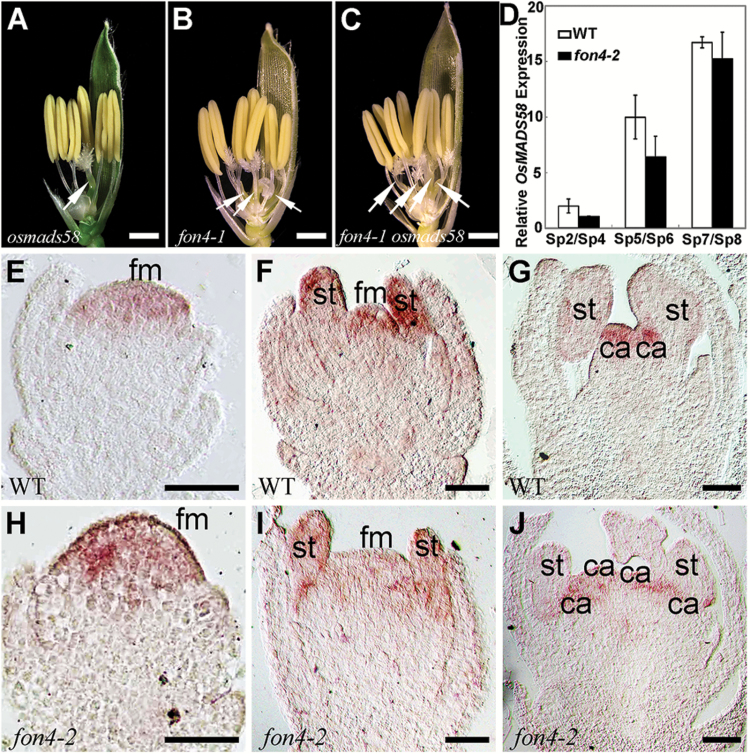
Phenotype of *fon4-1 osmads58*. (A–C) The flowers of *osmads58* (A), *fon4-1* (B), and *fon4-1 osmads58* (C) with the removal of the lemma and half the palea. Arrows indicate pistils. (D) qRT–PCR analysis of *OsMADS58* expression in wild-type and *fon4-2* flowers. (E–J) *OsMADS58* expression pattern in wild-type (E–G) and *fon4-2* (H–J) flowers. ca, carpel; fm, floral meristem; st, stamen. Scale bars=1 mm (A–C) and 50 μm (E–J).

In rice, another C-class gene, *OsMADS58*, has a partially divergent function from *OsMADS3* (Yamaguchi *et al.*, 2006; Dreni *et al.*, 2011). An insertional mutant of *OsMADS58* carrying a *dSpm* element in the second intron displayed a normal phenotype, except that few flowers generated bifurcated stigmas (Fig. 4A; Table 1) (Dreni *et al.*, 2011). We combined *osmads58* and *fon4-1*, another allele of *FON4*, which has a deletion of ~200 kb (Chu *et al.*, 2006). *fon4-1 osmads58* formed three outer floral whorls similar to those of *fon4-1*, whereas the number of pistils in the double mutant was slightly increased compared with that of *fon4-1* (Fig. 4B, C; Table 1), suggesting that *osmads58* enhances the defect of *fon4* in FM activity, similar to that of *osmads3*. In the wild type, *OsMADS58* expression is first detectable when *OsMADS3* transcripts start to accumulate, but it is uniformly distributed in the FM. After the stamen primordia initiated, *OsMADS58* was persistently expressed in the developing inner two whorls, displaying an expression profile similar to that of *OsMADS3* (Dreni *et al.*, 2011). Although the FM of *fon4* mutants appeared larger than that of the wild type, leading to more carpel-like organs at the late stage (Chu *et al.*, 2006), our expression analysis showed that the *OsMADS58* expression level had no significant change in *fon4-2* (Fig. 4D–J; Supplementary Fig. S1). Based on these results, we concluded that *FON4* synergistically interacts with C-class genes *OsMADS3* and *OsMADS58* in the regulation of reproductive organ identity and FM determinacy.

### *FON4* and *OsMADS13* synergistically specify FM determinacy

**Fig. 5.**
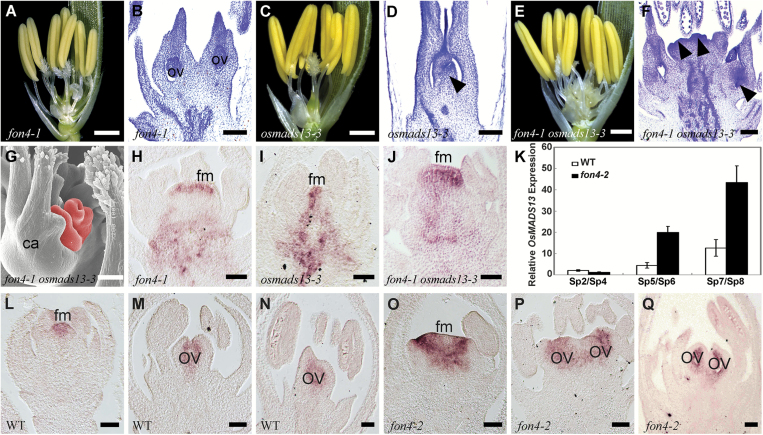
Phenotype of *fon4-1 osmads13-3*. (A, C, E) The flowers of *fon4-1* (A), *osmads13-3* (C), and *fon4-1 osmads13-3* (E) with the removal of the lemma and half the palea. (B, D, F) Longitudinal section of *fon4-1* (B), *osmads13-3* (D), and *fon4-1 osmads13-3* (F) stained with 0.1% Toluidine blue showing carpel and ovule development. The arrowheads in (D) and (F) indicate the carpelloid structures converted from the ovules. (G) SEM image of carpelloid structures (red region) in *fon4-1 osmads13-3*. (H–J) *OSH1* expression in *fon4-1* (H), *osmads13-3* (I), and *fon4-1 osmads13-3* (J). (K) qRT–PCR analysis of *OsMADS13* expression in wild-type and *fon4-2* flowers. (L–Q) *OsMADS13* expression pattern in wild-type (L–N) and *fon4-2* (O–Q) flowers. ca, carpel; fm, floral meristem; ov, ovule. Scale bars=1 mm (A, C, E), 100 μm (B, D, F, G), and 50 μm (H–J, L–Q).

The rice D-class gene *OsMADS13* regulates ovule identity and specifies FM termination redundantly with *OsMADS3* and *OsMADS58* (Dreni *et al.*, 2007, 2011; Li *et al.*, 2011*b*). In *osmads13-3*, the ovules were homeotically transformed into carpelloid structures, and 3–4 stigmas formed in some flowers (Fig. 5C, D; Table 1) (Li *et al.*, 2011*b*). *fon4-1 osmads13-3* displayed changes in the number of floral organs of the outer three whorls, which may be attributable to the mutation in *fon4-1*, but the number of carpelloid structures in the double mutant was greatly increased in comparison with *fon4-1* (Fig. 5A, B, E–G; Table 1). The expression domain of *OSH1* in *fon4-1 osmads13-3* appeared wider than that in either single mutant at the stage when the FM terminated in the wild type (Fig. 5H–J). Accordingly, we propose that *FON4* and *OsMADS13* synergistically specify FM determinacy. Notably, the qRT–PCR result showed that the *OsMADS13* expression level was higher in the *fon4*-2 mutant than in the wild type (Fig. 5K). In addition, the expression region of *OsMADS13* became much larger in *fon4*-2 (Fig. 5L–Q; Supplementary Fig. S1) (Lopez-Dee *et al.*, 1999; Dreni *et al.*, 2007). One possible explanation is that *FON4* may repress *OsMADS13* expression, and the increased expression of *OsMADS13* could represent a molecular response which compensates the loss of *fon4-2*, to alleviate the expansion of the meristem. Therefore, we concluded that *OsMADS13* and *FON4* may regulate each other at the transcriptional level.

### *FON4* and *OsMADS1* synergistically maintain FM identity

**Fig. 6.**
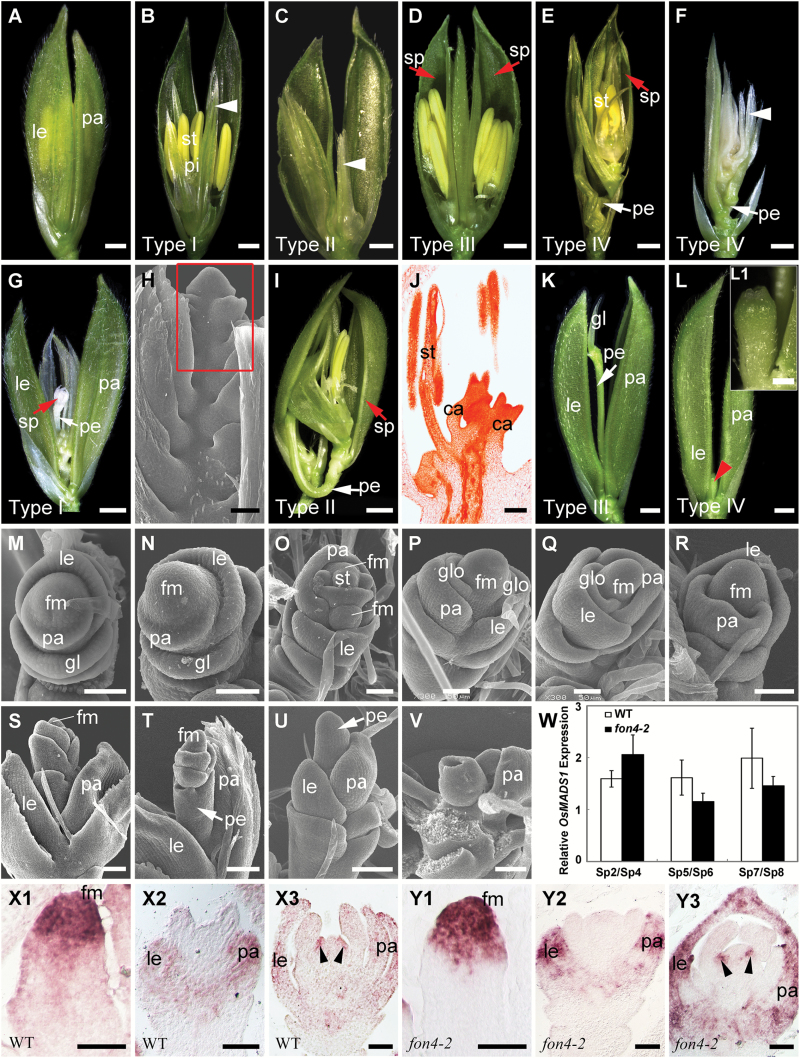
Phenotype of *fon4-2 osmads1-z.* (A) *osmads1-z* flower with leaf-like lemma and palea. (B–D) *osmads1-z* flowers of type I (B), type II (C), and type III (D). A half of a leafy lemma and palea were removed, and arrowheads in (B) and (C) indicate glume-like organs. (E, F) Type IV flower of *osmads1-z* with a new spikelet in the center. The spikelet in (E) has stamens and carpels in the inner whorls, whereas the spikelet in (F) only contains repetitious glume-like organs (arrowheads). A half of a leafy lemma and palea of primary and secondary flowers were removed in (E), and the whole leafy lemma and palea were removed in (F). (G) Type I flower of *fon4-2 osmads1-z* generating a new indeterminate composed of only glume-like organs. (H) SEM image of that in (G). The red square indicates the indeterminate FM. (I) Type II flower of *fon4-2 osmads1-z* generating a new determinate spikelet with stamens and carpels in the inner whorls. (J) Longitudinal section of the spikelet in (I). (K) Type III flower of *fon4-2 osmads1-z* containing only a glume on the pedicel. (L) Type IV flower of *fon4-2 osmads1-z* with an undefined structure (red arrowhead) inside the flower. (L1) A close-up view of the undefined structure in (L). (M, N) SEM images of *osmads1-z* (M) and *fon4-2 osmads1-z* (N) at the stage when the glume, lemma, and palea primordia are formed. (O) SEM image of the *osmads1-z* flower at the stage when stamen primordia initiate. (P–V) SEM images of *fon4-2 osmads1-z* flowers. (W) qRT–PCR analysis of *OsMADS1* expression in wild-type and *fon4-2* flowers. (X1–Y3) *OsMADS1* expression pattern in wild-type (X1–X3) and *fon4-2* (Y1–Y3) flowers. Arrowheads in (X3) and (Y3) indicate carpels. sp, spikelet; fm, floral meristem; gl, glume; le, lemma; pa, palea; pe, pedicel; glo, glume-like organ; st, stamen. Scale bars=1 mm (A–G, I, K, L), 500 μm (L1), 100 μm (H, J, S–V), and 50 μm (M–R, X1–Y3).

*OsMADS1*, an E-class gene, has been shown to be involved in promoting FM identity and specifying floral organ identity (Jeon *et al.*, 2000*a*; Prasad *et al.*, 2001, 2005; Agrawal *et al.*, 2005; Chen *et al.*, 2006; Gao *et al.*, 2010; Li *et al.*, 2010; Khanday *et al.*, 2013; Hu *et al.*, 2015). As we described previously (Gao *et al.*, 2010; Hu *et al.*, 2015), the *osmads1-z* allele produced a leafy lemma and palea, showing open flowers with four types of flower patterning for the inner whorls (Fig. 6A–F): type I (69%, *n*=320) with a change in the number of stamens and pistils (Fig. 6B); type II (16%, *n*=320) with glume-like organs in place of inner floral organs (Fig. 6C); type III (9%, *n*=320) with twin flowers in each spikelet (Fig. 6D); and type IV (6%, *n*=320) with a new spikelet along a pedicel comprised of stamens and carpels or only glume-like organs in the inner whorls (Fig. 6E, F). Despite the fact that *fon4-2 osmads1-z* produced abnormal lemma and palea resembling those of *osmads1-z*, the double mutant lacked the inner floral organs; instead, they generated a new spikelet along with the long pedicel or an undefined organ in the inner whorls (Fig. 6G–L). *fon4-2 osmads1-z* flowers were mainly classified into four types: type I showing an indeterminate spikelet composed of repetitious glume-like organs (44%, *n*=352) (Fig. 6G, H); type II displaying a determinate spikelet containing stamens and carpels in the inner whorls (22%, *n*=352) (Fig. 6I, J); type III bearing glumes or no organs along with the pedicel (13%, *n*=352) (Fig. 6K); and type IV having an undefined organ in the center (22%, *n*=352) (Fig. 6L, L1). Overall, *fon4-2* enhanced the defect of *osmads1-z* in FM identity, and thus we inferred that *FON4* and *OsMADS1* synergistically maintain FM identity. Detailed observation showed that *fon4-2 osmads1-z* flowers failed to generate stamen primordia after the lemma and palea were formed in *osmads1-z* flowers. Instead, the double mutant either continued to produce glume-like organs at the flank of the FM or remained an indeterminate meristem (Fig. 6M–R), causing the formation of different types of spikelets inside the lemma and palea (Fig. 6S–V). Further expression analysis showed that no obvious expression change of *OsMADS1* was observed in *fon4-2* at various stages during flower development (Fig. 6W–Y3; Supplementary Fig. S1).

## Discussion

The FM is determinate, and its activity is maintained until all the floral organs are formed (Steeves and Sussex, 1989). On the other hand, the rice FM still persists after the carpel develops and, instead, it is completely consumed when the ovule forms (Dreni *et al.*, 2007). In rice, *FON1* and *FON4* are the orthologs of *CLV1* and *CLV3*, respectively (Suzaki *et al.*, 2004, 2006; Chu *et al.*, 2006; Chu and Zhang, 2007), but which factor(s) is(are) functionally similar to *WUS* is still unknown. It has been indicated that rice C-class genes *OsMADS3* and *OsMADS58*, and the D-class gene *OsMADS13* redundantly regulate FM determinacy (Dreni *et al.*, 2011; Li *et al.*, 2011*b*). In this work, we have investigated the genetic relationship between *FON4* and floral homeotic genes. *FON4* showed an additive interaction with *OsMADS15* and a synergistic interaction with *OsMADS16* in the control of the organ number of whorl 2. On the other hand, the phenotypic analysis of double mutant combinations of *fon4* with *osmads3*, *osmads58*, *osmads13*, and *osmads1* individually indicates that *FON4* and these genes act in parallel pathways that converge on a common process of FM activity control. However, it is unknown where and how these pathways converge.

### Conserved and diversified genetic control of floral organ number and identity

The FM in angiosperms develops into floral organs whose numbers, positions, and identities lead to the diversification of flowers. A lot of genes that regulate floral organ number have been identified in Arabidopsis. For example, mutations in *CLV* genes, *WIGGUM* (*WIG*), and *ULTRAPETALA1* (*ULT1*) cause an increase in floral organ number (Leyser and Furner, 1992; Clark *et al.*, 1993, 1995, 1997; Kayes and Clark, 1998; Running *et al.*, 1998; Fletcher *et al.*, 1999; Jeong *et al.*, 1999; Ziegelhoffer *et al.*, 2000; Fletcher, 2001; Carles *et al.*, 2005), whereas mutations in *TOUSLED* (*TSL*), *REVOLUTA* (*REV*), *FASCIATA* (*FAS*), and *WUS* result in fewer floral organs (Leyser and Furner, 1992;  Roe *et al.*, 1993, 1997; Talbert *et al.*, 1995; Mayer *et al.*, 1998; Kaya *et al.*, 2001). In these mutants, the changes in organ number are related to FM size during the time of organ primordia initiation. After organ primordia initiation at the correct position, floral homeotic genes sequentially specify organ identities. Therefore, the mechanisms of floral organ number regulation and determination of floral organ identity seem to be two separate processes. Clark and co-workers reported that double mutant combinations of *clv1* with *ap2*, *ap3*, *pistillata* (*pi*), and *ag* were all additive in phenotype, indicating that *CLV1* regulates the FM structure independently of these homeotic gene functions. The double mutant with *ap1* displayed the enhanced defects in FM identity. Furthermore, expression patterns of *AG* and *AP1* were altered in the *clv1* mutant.

In this study, we analyze the phenotypes of the double mutant combinations of the *CLV3* ortholog *FON4* and rice floral homeotic genes. Some differences are discovered in comparison with the corresponding double mutants of *clv1.* First of all, the *fon4 dep* double mutant shows an additive phenotype (Fig. 1A–H), whereas *clv1 ap1* exhibits an enhancement in FM identity defects. Occasionally, a new inflorescence meristem is generated in the center of *clv1 ap1* flowers that was not observed in the single mutants. This difference might result from the distinct effects of *ap1* and *dep* mutations on FM activity. Unlike the *ap1* mutation which affects sepal and petal development, and causes a partial transformation of a floral meristem into an inflorescence meristem (Irish and Sussex, 1990; Mandel *et al.*, 1992), the *dep* mutant only shows a stable degenerative palea phenotype (Wang *et al.*, 2010). In the rice genome, there are four orthologs of Arabidopsis *AP1*, namely *OsMADS14*, *OsMADS15*, *OsMADS18*, and *OsMADS20* (Litt and Irish, 2003; Kater *et al.*, 2006). We speculate that these *AP1*-like genes work redundantly, and thus neither *dep* nor *fon4 dep* shows abnormality in FM identity. Secondly, the double mutants of *FON4* with *AG*-like genes *OsMADS3* or *OsMADS58* display the enhanced defect in FM size and determinacy, and the *fon4* mutation enhances the stamen identity defect of the *osmads3-4* allele (Figs 3A–P, 4 A–C), which has not been observed in the *clv1 ag* double mutant (Clark *et al.*, 1993). However, since the reported double mutant contained a strong *ag* allele, consisting of only sepals and petals, it is unknown whether the *clv* mutants can also enhance the *ag* phenotype of floral organ identity transformation. Additionally, the expression patterns of *OsMADS15*, *OsMADS3*, and *OsMADS58* in the *fon4* mutant mirror those in the wild type (Figs 1I–O, 3Q–W, 4D–J). In contrast*, AP1* and *AG* are altered in *clv1* flowers in Arabidopsis (Clark *et al.*, 1993). In the wild type, *AG* was uniformly expressed throughout the FM at the early stage (Drews *et al.*, 1991). However, the *clv1* mutants lack *AG* expression in the very center of the FM (Clark *et al.*, 1993). The absence of detectable *AG* expression in the center of the ﬂower possibly resulted in continued proliferation of these central cells.

### *FON4* regulates FM activity in parallel with C, D, and E genes

*FON4* is responsible for the regulation of FM size. The *fon4* mutation causes an increase in the number of all floral organs owing to the enlarged FM (Chu *et al.*, 2006; Suzaki *et al.*, 2006). In addition, *FON4* also controls FM determinacy at the later stage. In the wild type, the FM activity terminates after the carpel develops in whorl 4. Conversely, the FM in the *fon4* mutant still remains and the meristem marker gene *OSH1* continues to be expressed even after a number of carpels have been formed (Fig. 3N) (Chu *et al.*, 2006; Suzaki *et al.*, 2006). Among the floral homeotic genes selected in this study, *OsMADS15* plays a minor role in FM determinacy. The *fon4-2 dep* double mutant displays an additive phenotype, indicating that *FON4* acts independently of *OsMADS15* (Fig. 1A–H). *OsMADS16* has been reported to have a function in FM determinacy (Li *et al.*, 2011*a*; Yun *et al.*, 2013). *fon4-2 spw1-1* displays an enhanced effect in the organ number of whorl 2, although this change in the internal whorls is not as clear (Fig. 2A, B; Table 1), since it is difficult to measure carpel number directly in the mutant where multiple carpels could form one fused gynoecium, and not all of them will produce a stigma. *OsMADS3*, *OsMADS58*, and *OsMADS13* are instead important for FM determinacy, and FM determinacy is enhanced in all the double mutant combinations of *fon4* with these genes (Figs 3A–P, 4A–C, 5A–J). The altered phyllotaxy (from whorled to distichous) seen occasionally in *osmads1-z* (Fig. 6F) is significantly enhanced in *fon4-2 osmads1-z* (Fig. 6G, H), suggesting that *FON4* acts synergistically with *OsMADS1* to promote the transition from spikelet meristem to floral meristem, and to maintain FM identity. Intriguingly, these data suggest that there could be feedback between FM size and FM identity, and that it is difficult to maintain FM identity as FM size abnormally increases. It is possible that *fon4-2* enhances the loss of stamen identity in *osmads3-4* (Fig. 3A–C) through a similar mechanism. Furthermore, the expression patterns of *OsMADS3*, *OsMADS58*, and *OsMADS1* have no obvious change in the *fon4* mutants (Figs 3Q–W, 4D–J, 6W–Y3). Therefore, it seems that *FON4* genetically controls FM activity together with *OsMADS3*, *OsMADS58*, and *OsMADS1*, but without regulating each other’s transcription. The *OsMADS13* expression level is up-regulated in the *fon4* mutant, though its expression remains restricted to the FM and ovule primordia (Fig. 5K–Q), suggesting that *FON4* may indirectly repress *OsMADS13* expression. A feedback loop could exist so that loss of *FON4* is compensated by an increased expression of *OsMADS13*, which contributes to reducing meristem expansion.

### *FON4* enhances the function of C, D, and E genes at different stages

**Fig. 7.**
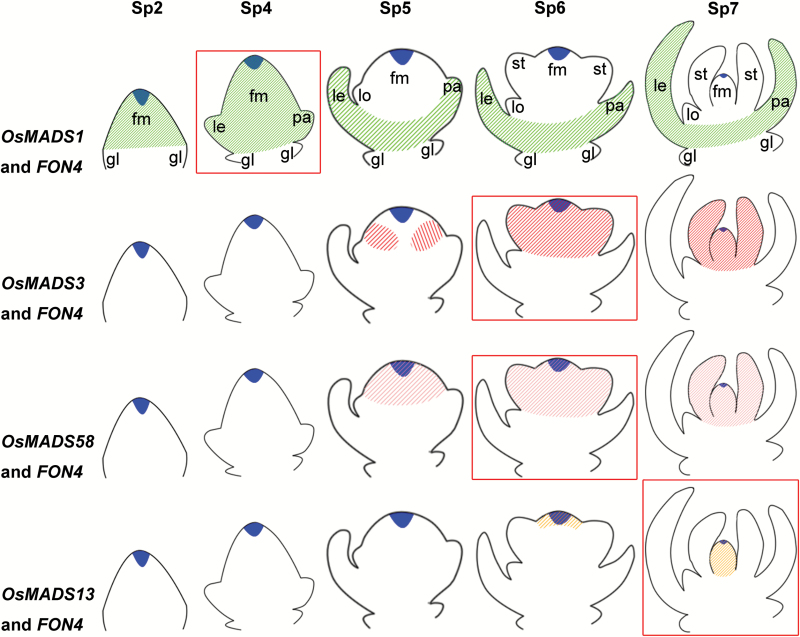
Comparison of the expression pattern of *FON4* and floral homeotic genes from stage Sp2 to Sp7. Blue indicates the expression region of *FON4*, and the green, red, pink, and orange oblique lines indicate the expression region of *OsMADS1*, *OsMADS3*, *OsMADS58*, and *OsMADS13*, respectively. At the stages (red square) when apparent abnormalities are observed in the double mutants, the expression region of *FON4* partially overlaps with these genes in the floral meristem. fm, floral meristem; gl, glume; le, lemma; lo, lodicule; pa, palea; st, stamen.

In rice flower development, *FON4* is persistently expressed in the central region of the FM apex until the carpel is formed (Fig. 7) (Chu *et al.*, 2006; Suzaki *et al.*, 2006). We compared the expression regions of *FON4* and *OsMADS1*, *OsMADS3*, *OsMADS58*, and *OsMADS13* in the wild type at the stage when the double mutants started to display the obviously enhanced defects (Fig. 7). *OsMADS1* expression is first detectable in the incipient FM, and later it is restricted in the developing lemma/palea and carpel (Prasad *et al.*, 2001, 2005). *fon4-2 osmads1-z* displays apparent abnormality after the formation of the lemma and palea primordia (stage Sp4; Fig. 6M–R). At this stage, *OsMADS1* is expressed in the lemma, palea, and FM in the wild type, encompassing the expression region of *FON4*. *OsMADS3* is initially expressed in the founder cells recruited to stamen primordia, then its expression is retained in the two inner whorls (Dreni *et al.*, 2011). When the stamen primordia emerge in whorl 3 (stage Sp6; Fig. 3E–J), *fon4-2 osmads3-4* shows an enhanced phenotype. The expression of *OsMADS3* at this stage is observed in stamen primordia and in the FM, including the *FON4* expression region. The *fon4-1 osmads58* double mutant has enhanced defects in FM determinacy similar to *fon4 osmads3*. *OsMADS13* is expressed in the FM before the differentiation of carpel and ovule primordia. Subsequently, it continues to be expressed in the ovule primordium and in the inner layer of the carpel wall (Lopez-Dee *et al.*, 1999; Dreni *et al.*, 2007, 2011). In the *fon4-1 osmads13-3* double mutant, the *OSH1* expression region is much larger than that of either single mutant at the stage when the stamen differentiates into anther and filament, whereas carpel and ovule primordia have not emerged (stage Sp7; Fig. 5H–J). At this stage, the expression region of *OsMADS13* includes that of *FON4.* Taken together, these data suggest that *FON4* enhances the function of *OsMADS1*, *OsMADS3*, *OsMADS58*, and *OsMADS13* at different stages, and that the expression region of *FON4* partially overlaps with these genes in the FM.

In Arabidopsis, *AG* and *CLV* pathways appear to mediate *WUS* repression in partially independent ways, since the phenotype of the *clv1 ag* double mutant was substantially additive and the *WUS* expression domain was larger in *ag clv1* double mutants than in *ag* single mutants. In our study, *fon4* synergistically interacts with *osmads3*, *osmads58*, *osmads13*, and *osmads1* in FM activity; therefore, we speculate that *FON4* and these genes regulate a common target gene, similarly to *WUS* regulation, through parallel pathways at the stage when the significantly enhanced defects are observed. However, such a factor has not been identified in rice. Phylogenetic analysis revealed that rice contained a single *WUS* ortholog *OsWUS* (also called *MONOCULM3*, *MOC3* and *TILLERS ABSENT1*, *TAB1*) (Nardmann and Werr, 2006; Zhang *et al.*, 2010; Lu *et al.*, 2015; Tanaka *et al.*, 2015). Nardmann and Werr (2006) reported that the expression pattern of *OsWUS* was markedly different from that of *WUS* in Arabidopsis because it was not expressed in the OC of shoot meristems. Later studies showed that *OsWUS* transcript was detected in the pre-meristem zone during axillary meristem development (Tanaka *et al.*, 2015). In contrast to the Arabidopsis *wus* mutant, which produced numerous adventitious shoots, mutations in *OsWUS* resulted in no tiller formation (Lu *et al.*, 2015; Tanaka *et al.*, 2015), demonstrating that *WUS* may have a diverged function between rice and Arabidopsis. Moreover, *OsWUS* is expressed in the apical region of branch and spikelet meristems (Tanaka *et al.*, 2015). The *moc3-1* allele seemed to be female sterile, whereas the *tab1-1* allele showed reduced spikelet number and abnormal spikelet structure (Lu *et al.*, 2015; Tanaka *et al.*, 2015). Another *WOX* gene, *OsWOX4*, is required for the maintenance of the vegetative meristem and is negatively regulated by *FON2-LIKE CLE PROTEIN1* (*FCP1*) (Ohmori *et al.*, 2013). Although *OsWOX4* expression is detected in all reproductive meristems, its role in inflorescence and spikelet development is still unknown. Therefore, the functional analysis of CLV–WUS-like pathways in rice will provide clues about their conservation and diversification in meristem maintenance between grasses and eudicots.

## Supplementary data

Supplementary data are available at *JXB* online.

Fig. S1. Sense probes were used as negative controls for *in situ* hybridization experiments.

Table S1. Primers used in this study.

Table S2. Floral meristem sizes in the wild type and mutants.

## Supplementary Material

supplementary_figure_S1_tables_S1_S2
