# Supplementary material for: Interactions between FLORAL ORGAN NUMBER4 and floral homeotic genes in regulating rice flower development
Source: J Exp Bot. 2017 Jan 20;68(3):483–98. doi: 10.1093/jxb/erw459 (PMC6055531; doi:10.1093/jxb/erw459)
Supplement: supplementary_figure_S1_tables_S1_S2 [file erw459_suppl_supplementary_figure_s1_tables_s1_s2.pdf]

Supplementary Figure S1. Sense probes were used as negative controls for *in situ* hybridization experiments

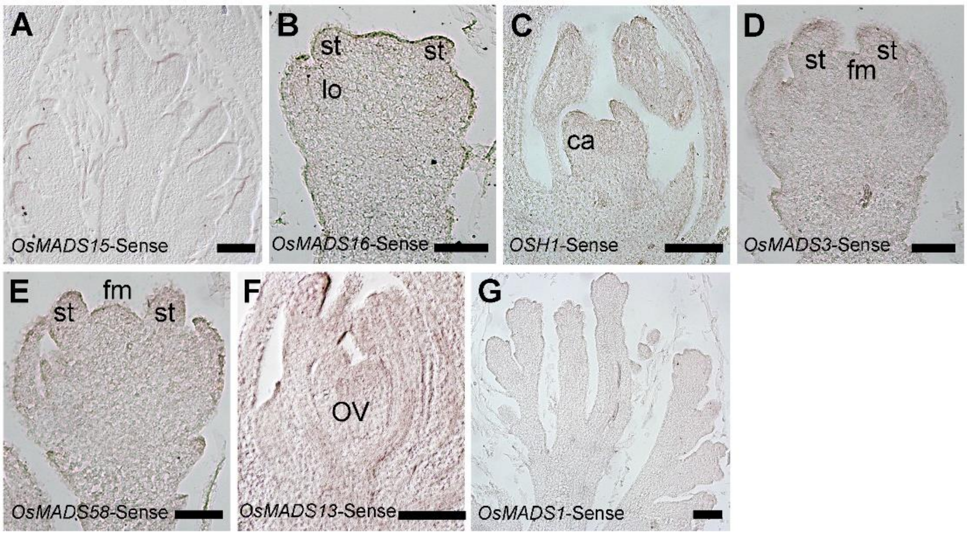

(A-G) Sense probes for *OsMADS15* (A), *OsMADS16* (B), *OSH1* (C), *OsMADS3* (D), *OsMADS58* (E), *OsMADS13* (F), and *OsMADS1* (G). Only background signals were observed when sense probes were used. ca, carpel; fm, floral meristem; lo, lodicule; st, stamen. Scale bars = 100 μm (A, C, F, G) and 50 μm (B, D, E).

Table S1. Primers used in this study

Name	Sequence (5’→3’)	Purpose
FON4-2F	GGTACGTCGTAGGAGCTTCA	*fon4-2* genotyping
FON4-2R	CAGCAATGCATGCAAACTAC	
FON4-1F	AGACGTACAGTGTCCGTTCCA	*fon4-1* genotyping
FON4-1R	CGCACCTACTCTACTAACCCTACA	
FON4-1-mR	CCACCTACCTCCACTTCTCCA	
MADS15-F	TCCCTCTCCTCCTCCTCTTCTTCTT	*dep* genotyping
MADS15-R	GGAATCGATCAGGCTATAGGTGTAC	
MADS16TPF	ATCTTTGACCGCTACCAGCAAG	*spw1-1* genotyping
MADS16TPR	ATTTCCACACATGCAAGAAGCC	
MADS3TPF	ACCAGCAGGAGTCCTCCAAAC	*osmads3-4* genotyping
MADS3TPR	CAACTTCAGCATATAACAGCTCATTC	
Osp196	GTGTGGGGTTTTGGCCGACAC	*osmads58* genotyping
Osp201	GATGATGGCCATGATGTCTCC	
Osp202	ATGCATGTACATGCTCATGCTTG	
MADS13TPF	AGATGCTGCAAAACACCAACAA	*osmads13-3* genotyping
MADS13TPR	TGATCTCTGAAGCCAGCAGTTC	
MADS1-F	TACGATCAGGTAGCCAAACCAC	*osmads1-z* genotyping
MADS1-900R	ACATGATCAAAGTGAGTTCTGCGT	
MADS1-700R	ATTTCTGCCTATTACACGGATGG	
MAS15-qF	TTGGTGCCATGAATACAGGA	*OsMADS15* qRT-PCR
MAS15-qR	CTCGGAAATGGACTCAAGCA	
MAD16-qF	TCAAGGACATCAACCGCAACCTG	*OsMADS16* qRT-PCR
MAD16-qR	ATGATACTTCCTGTGGCGAACCTC	
RT973	GGGATTCTATCAACACCATGAG	*OsMADS3* qRT-PCR
RT974	CTCAACTTCAGCATATAACAGC	
RT975	CTGCTAAGCTGAAGCAACAG	*OsMADS58* qRT-PCR
RT976	CTTCCAGCTGCTTAAGTTCTC	
RT977	CTGGTTGGCGATAATGTGAG	*OsMADS13* qRT-PCR
RT978	CCAGCAGTTCATTCTTCCTG	
MADS1-qF	ATCACCATCAGGGTCTTCTC	*OsMADS1* qRT-PCR
MADS1-qR	CAACCATGTCTGCTGCTTCA	
ACTIN-F	CCTTCAACACCCCTGCTATG	*ACTIN* qRT-PCR
ACTIN-R	CAATGCCAGGGAACATAGTG	

Table S2. Floral meristem sizes in the wild type and mutants.
The average size is shown as mean ± SD (standard deviation).

**Genotype**	**Length (μm)**	**Width (μm)**	**No. of spikelets measured**
Wild type	45.81±8.96	35.19±5.33	10
fon4-2	97.56±9.77	74.81±7.11	14
osmads3-4	62.97±5.14	45.95±8.73	9
fon4-2 osmads3-4	129.56±24.57	69.17±15.47	10
Floral meristem sizes were examined by scanning electron microscopy (SEM) when the stamen primordia initiated in whorl 3. The Length and width of floral meristem were longest distance along the longitudinal direction and transverse direction, respectively.			
